# Síndrome Cardiorrenal Aguda: Qual Critério Diagnóstico Utilizar e sua Importância para o Prognóstico?

**DOI:** 10.36660/abc.20190207

**Published:** 2020-07-28

**Authors:** Andréa de Melo Leite, Bruno Ferraz de Oliveira Gomes, André Casarsa Marques, João Luiz Fernandes Petriz, Denilson Campos de Albuquerque, Pedro Pimenta de Mello Spineti, Antonio José Lagoeiro Jorge, Humberto Villacorta, Wolney de Andrade Martins

**Affiliations:** 1 Universidade Federal Fluminense Faculdade de Medicina NiteróiRJ Brasil Universidade Federal Fluminense - Faculdade de Medicina - Pós-graduação em Ciências Cardiovasculares, Niterói, RJ – Brasil; 2 Rede D’Or São Luiz Rio de JaneiroRJ Brasil Rede D’Or São Luiz, Rio de Janeiro, RJ – Brasil; 3 Instituto D’Or de Pesquisa e Ensino Rio de JaneiroRJ Brasil Instituto D’Or de Pesquisa e Ensino, Rio de Janeiro, RJ – Brasil; 4 Universidade do Estado do Rio de Janeiro Rio de JaneiroRJ Brasil Universidade do Estado do Rio de Janeiro, Rio de Janeiro, RJ – Brasil

**Keywords:** Síndrome Cardiorrenal, Insuficiência Renal, Creatinina, Prognóstico, Insuficiência Cardíaca, Revisão Sistemática

## Abstract

A indefinição de critérios diagnósticos para síndrome cardiorrenal aguda (SCRA) impacta em diferentes resultados prognósticos. Objetivou-se avaliar os critérios diagnósticos da SCRA e o impacto no prognóstico. Procedeu-se à revisão sistemática utilizando-se a metodologia PRISMA e os critérios PICO nas bases MEDLINE, EMBASE e LILACS. A pesquisa incluiu artigos originais do tipo ensaio clínico, coorte, caso-controle e meta-análises publicados no período de janeiro de 1998 até junho de 2018. Não foi encontrada na literatura nem nas diretrizes de insuficiência cardíaca uma definição clara dos critérios diagnósticos da SCRA. O critério diagnóstico mais comumente utilizado é o aumento da creatinina sérica de pelo menos 0,3 mg/dl em relação à basal. Entretanto, existem controvérsias na definição de creatinina basal e de qual deveria ser a creatinina sérica de referência dos pacientes críticos. Esta revisão sistemática sugere que os critérios de SCRA devem ser revistos para que se inclua o diagnóstico de SCRA na admissão hospitalar. A creatinina sérica de referência deve refletir a função renal basal antes do início da injúria renal aguda.

## Introdução

A insuficiência cardíaca (IC) é um desafio clínico e um problema epidemiológico em progressão em todo o mundo, com elevada morbimortalidade.^1^ No estudo ARIC,^2^ as taxas de letalidade em 30 dias, 1 ano e 5 anos após hospitalização por IC foram 10,4%, 22,0% e 42,3%, respectivamente. O I Registro Brasileiro de Insuficiência Cardíaca (BREATHE),^3^ estudo observacional com 1263 pacientes de diferentes regiões do Brasil, mostrou uma mortalidade hospitalar de 12,6%.

A síndrome cardiorrenal, definida como um processo de injúria renal ocasionada por IC, foi descrita pela primeira vez em 1951^4^ e categorizada no ano de 2008 em cinco subtipos ( Tabela 1 ).^5^ A síndrome cardiorrenal tipo 1, ou síndrome cardiorrenal aguda (SCRA), caracteriza-se pela injúria renal aguda (IRA) ocasionada por IC descompensada (ICD). Alguns autores referem-se à SCRA por piora da função renal no paciente com IC ( *Worsening Renal Function - WRF* ). Este fenômeno é uma condição frequente, acometendo 11% a 40% das internações por ICD.^6 , 7^

**Tabela 1:** – Subtipos de síndrome cardiorrenal

Tipo	Nome	Mecanismo
1	SCR aguda	IRA induzida por disfunção cardíaca aguda
2	SCR crônica	IRA progressiva secundária à IC crônica
3	Síndrome renocárdica aguda	IC aguda precipitada por IRA
4	Síndrome renocárdica crônica	IC secundária à IRC
5	SCR secundária	Disfunção miocárdica e renal por doenças sistêmicas

*SCR: síndrome cardiorrenal; IRA: injúria renal aguda; IRC: insuficiência renal crônica; IC: insuficiência cardíaca. Adaptado de Di Lullo et al.^40^*

A piora da função renal é avaliada pelo aumento da creatinina sérica, geralmente >26,5 μmol/l, equivalente a 0,3 mg/dl, e/ou aumento de 25% na creatinina ou queda de 20% na taxa de filtração glomerular (TFG).^8^ O critério de aumento absoluto da creatinina em 0,3 mg/dl tem sido adotado pela maioria dos autores como ponto de corte arbitrado para definir a SCRA.

O registro norte-americano ADHERE^9^ é um estudo observacional com mais de 100 mil pacientes admitidos com ICD. Nesse registro, aproximadamente 35% dos pacientes apresentaram disfunção renal moderada a grave.

A piora da função renal ocorre em 30% a 50% dos casos de ICD, dependendo da definição utilizada, e está associada com maior tempo de hospitalização, despesas de saúde elevadas e maior mortalidade.^10 - 14^ Entretanto, a ausência de uma definição consensual para a SCRA contribui para a falta de clareza em seu diagnóstico e tratamento.^15^ Um desafio é a escolha de qual creatinina sérica utilizar para determinar o cumprimento dos critérios diagnósticos da SCRA. Idealmente, a creatinina sérica de referência deve refletir a função renal basal antes do início da IRA. Na maioria das vezes, essa informação não está disponível, levando ao uso de valores de referência substitutos que podem influenciar o diagnóstico e a gravidade da SCRA.^16^

## Métodos

Esta revisão foi conduzida conforme a metodologia *Preferred Reporting Items for Systematic Reviews and Meta-Analyses* (PRISMA).^17^ A pesquisa incluiu artigos originais do tipo ensaio clínico, coorte, caso-controle e meta-análises publicados no período de janeiro de 1998 até junho de 2018, com textos na íntegra, em inglês, espanhol e português, nas bases de dados MEDLINE, EMBASE e LILACS. A pesquisa foi realizada com os seguintes descritores: ( *cardiorenal syndrome* ) OR *(worsening renal function)* AND ( *heart failure* ) AND ( *diagnosis* ) AND ( *prognosis* ).

Este estudo baseou-se nos critérios PICO (População, Intervenção, Controle e Resultados). No presente estudo procedeu-se à revisão dos critérios diagnósticos da SCRA e sua implicação prognóstica nos desfechos mortalidade hospitalar e mortalidade após a alta hospitalar, assim como no tempo de hospitalização. Foram excluídos relatos de caso e modelos experimentais animais.

## Resultados

**Figura 1:**
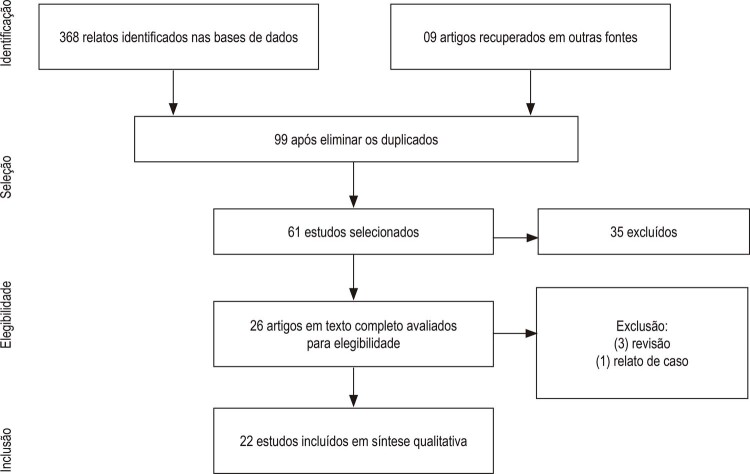
– *Fluxograma dos estudos avaliados (metodologia PRISMA^17^).*

Preencheram os critérios de pesquisa 368 resumos. Outros 9 artigos foram recuperados em outras fontes, sendo eliminados 278 resumos em duplicata nas bases de dados. Foram avaliados 99 resumos e selecionados 61, dos quais 35 foram excluídos por não atenderem aos critérios previamente estabelecidos (PICO). Portanto, 26 artigos de texto completo foram avaliados em relação à qualidade científica. Desses, 4 foram excluídos por não preencherem os critérios, resultando em um total de 22 artigos analisados ( Figura 1 ).

### Classificação temporal da síndrome cardiorrenal aguda

Estudos com acesso aos níveis pré-admissionais de creatinina sérica revelaram que a IRA esteve presente na admissão da emergência em cerca de um terço dos pacientes.^18^ Outros 50% dos pacientes apresentaram IRA nas primeiras 48 horas. Tayaka et al.,^19^ em estudo comparando as alterações da função renal até o quarto dia de internação e após o quinto dia de internação, observaram que os pacientes com lesão renal tardia apresentaram maior mortalidade em um ano após a alta hospitalar. Uma análise *post hoc* do estudo Pré-RELAX demonstrou que a queda na pressão arterial sistólica nas primeiras 48 horas de terapia vasodilatadora foi um preditor independente de IRA até o quinto dia de internação.^20^ Esses resultados sugerem que a redução na pressão de perfusão renal relacionada à terapia seja um dos principais mecanismos que levam à IRA nos primeiros dias de internação.

A SCRA pode ser classificada em intermitente ou persistente. A SCRA intermitente ocorre quando há flutuação dos níveis séricos de creatinina durante a internação com redução dos seus valores até o momento da alta hospitalar. A SCRA persistente ocorre quando a elevação da creatinina ou a queda na TFG persiste até o momento da alta hospitalar.^21 , 22^

### Incidência da síndrome cardiorrenal aguda

Existe uma grande variabilidade entre os estudos quanto à incidência de SCRA, cuja estimativa varia entre 19% e 45%. Essa variação pode ser atribuída aos diferentes critérios diagnósticos empregados, aos diferentes critérios de inclusão e exclusão, ao tamanho amostral de cada estudo, além das diferenças clínicas das populações estudadas. A maioria dos estudos refere-se a análises retrospectivas, secundárias e/ou *post hoc* de grandes bancos de dados^10 - 12 , 23 - 25^ ou a ensaios clínicos de terapia medicamentosa.^26^

### Critérios diagnósticos da síndrome cardiorrenal aguda

O primeiro trabalho a avaliar o impacto da piora da função renal em idosos internados com ICD, no ano de 2000, adotou como critério o aumento da creatinina em 0,3 mg/dl.^10^ Outro estudo demonstrou que aumentos de creatinina durante a internação da ordem de 0,1 mg/dl estavam associados a maior mortalidade hospitalar e tempo de internação. Nesse estudo, um aumento ≥0,3 mg/dl apresentou melhor sensibilidade e especificidade para prever óbito (81% e 62%, respectivamente) e tempo de internação maior que dez dias (64% e 65%, respectivamente).^11^

O aumento absoluto da creatinina em 0,3 mg/dl tem sido adotado pela maioria dos autores como critério para definir a SCRA.^27^ Entretanto, outros autores discordam desse critério por ele não considerar o grau de disfunção renal prévia, sugerindo o uso de uma das três diferentes classificações para definição de IRA.^28^ Essas definições não são específicas para ICD, tendo sido desenvolvidas para definição e classificação de IRA em diferentes cenários clínicos.

A classificação de RIFLE^29^ foi proposta em 2004 para definir e estratificar a gravidade da IRA. O acrônimo RIFLE foi criado a partir das primeiras letras de *Risk* (risco), *Injury* (injúria/lesão), *Failure* (falência), *Loss* (perda da função renal) e *End-stage renal disease* (doença renal em estágio terminal). A gravidade da IRA é determinada pelo parâmetro mais alterado (variação da creatinina, TFG e débito urinário).

A classificação proposta pela *Acute Kidney Injury Network* (AKIN)^30^ exclui os estágios de perda da função renal e doença renal terminal e os critérios baseados na TFG. O estadiamento deve ser realizado após correção da volemia do paciente, com exclusão da obstrução do trato urinário e considerando-se o critério mais alterado. Em 2012, a classificação proposta pelo grupo *Kidney Disease – Improving Global Outcomes* (KDIGO)^31^ trouxe algumas modificações à classificação anterior, adicionando para o terceiro estágio a redução da TFG para valores inferiores a 35ml/min/1,73m^2^ em pacientes menores de 18 anos e excluindo a necessidade de aumento mínimo de 0,5 mg/dl para pacientes com creatinina superior a 4 mg/dl.

Uma coorte que avaliou 637 internações por ICD com seguimento de 30 dias e 1 ano comparou o critério diagnóstico do aumento de creatinina ≥0,3 mg/dl com os critérios KDIGO, RIFLE e AKIN quanto à predição dos desfechos morte, reinternação por IC ou início de diálise. O desempenho dos quatro critérios foi semelhante na determinação de eventos adversos. Os benefícios do uso dos sistemas de classificação de IRA (RIFLE, AKIN, KDIGO) residem na possibilidade de identificar os pacientes com graus mais graves de IRA que passarão por eventos adversos em 30 dias e 1 ano.^32^ Os diferentes critérios diagnósticos de IRA encontrados na literatura estão resumidos na Tabela 2 .

**Tabela 2:** – Critérios usados pelas classificações RIFLE34, AKIN35, KDIGO36 e WRF11 para definir injúria renal aguda

Critérios	WRF	RIFLE		AKIN	KDIGO
Anos	2002	2004		2007	2012
Classificação	Aumento da CrS	Aumento da CrS	Queda na TFG	Aumento da CrS	Aumento da CrS
Estágio 1 / Risco	≥ 0,3 mg/dl	≥ 1,5x CrB	≥ 25%	> 1,5-1,9x CrB ou ≥ 0,3 mg/dl	≥ 1,5x CrB ou ≥ 0,3 mg/dl
Estágio 2 / Injúria	-	≥ 2x CrB	≥ 50%	> 2-2,9x CrB	≥ 2x CrB
Estágio 3 / Falência	-	≥ 3x CrB	≥ 75%	≥ 3x CrB	≥ 3x CrB
	-	Ou CrS ≥ 4mg/dl e aumento de 0,5mg/dl		Ou CrS ≥ 4mg/dL e aumento de 0,5mg/dl ou início de diálise	Ou CrS ≥ 4mg/dl
Período mínimo para que IRA ocorra	Aumento da CrS em qualquer momento da internação	Variações da CrS em 1-7 dias por mais de 24 h		Alterações agudas da sCr em 48 h durante a internação	Elevação ≥ 1,5x CrB em 7 dias, ou aumento mínimo de 0,3 mg/dl na CrS em 48 h

*WRF (worsening renal function), piora da função renal; IRA, injúria renal aguda; CrS: creatinina sérica; TFG: taxa de filtração glomerular; CrB: creatinina basal.*

*Fonte: Adaptada de Roy et al.^32^*

O critério diagnóstico mais comumente utilizado é o aumento da creatinina sérica de pelo menos 0,3 mg/dl ou pelo menos 25% nos primeiros cinco dias de internação, o que difere da definição atual do KDIGO para IRA.^33^ Além disso, a definição da piora da função renal não inclui IRA na admissão, que está associada a mortalidade e eventos cardiovasculares.^34^

Abordagens comuns para o diagnóstico de SCRA incluem o uso das seguintes referências de creatinina basal, a partir da qual o aumento de creatinina define essa síndrome: a) creatinina admissional; b) a menor creatinina durante a internação hospitalar; c) valores de creatinina sérica de outras internações; ou d) medidas ambulatoriais de creatininemia. Os critérios originais do RIFLE não especificaram como escolher a creatinina de referência, mas recomendaram a imputação de um valor calculado a partir de uma TFG estimada de 75ml/min/1,73m^2^. Abordagens alternativas incluem a avaliação da variação da creatinina nas primeiras 48 horas para reduzir a necessidade do valor pré-hospitalar (AKIN) ou a menor creatinina sérica da internação, quando ausente a medida ambulatorial de creatinina (KDIGO).^35^

Siew et al.,^36^ estudaram 4.863 pacientes hospitalizados e avaliaram três referências de creatinina basal: MDRD ( *Modification of Diet in Renal Disease* ), creatinina admissional e a menor creatinina da internação. O MDRD e o *nadir* superestimaram a incidência em pelo menos 50%, enquanto a taxa de internação foi subestimada em 46%. O uso da creatinina da admissão como referência tem a menor sensibilidade para o diagnóstico de IRA adquirida no hospital e não inclui o diagnóstico de IRA iniciada antes da chegada ao hospital. Alguns autores consideraram como referência a creatinina da pré-admissão hospitalar (ambulatorial) quando disponível, mas apenas alguns deles definiram o tempo de validade máximo da medida ambulatorial até a internação. Porém, o valor ambulatorial da creatinina poucas vezes está disponível.

O critério RIFLE^29^ não define especificamente qual seria a referência de creatinina basal. O critério de IRA mais recente, KDIGO, sugere como referência a menor creatinina sérica durante a internação hospitalar.^35^ Poucos estudos consideraram a função renal basal correlacionada com creatinina ascendente durante o episódio de IRA.

### O uso de biomarcadores na caracterização da síndrome cardiorrenal aguda

Apesar de a creatinina ser o pilar do diagnóstico da SCRA, ela apresenta limitações como marcador da função renal, especialmente em pacientes críticos. Seu nível sérico é influenciado por fatores como sexo, idade, peso e massa muscular. Além disso, a creatinina eleva-se somente 24 horas após um dano renal e sua concentração não aumenta significativamente até que cerca de metade da função renal esteja comprometida. Portanto, a creatinina é considerada um marcador lento de IRA.^37^ Existem controvérsias na definição de creatinina basal dos pacientes críticos, visto que nessa condição existem alterações na nutrição, perda de massa muscular e sobrecarga hídrica.

Biomarcadores de qualidade são aqueles que possuem aplicabilidade clínica, com papel reconhecido na fisiopatologia da síndrome. Há incentivo para a pesquisa de biomarcadores mais fidedignos para o diagnóstico precoce da SCRA. A molécula de lesão renal-1 (KIM-1), a lipocalina associada à gelatinase de neutrófilos (NGAL), a interleucina 18 (IL-18), e a cistatina C (Cys-C) são alguns dos novos marcadores de lesão renal alvos de estudos. No entanto, nenhum dos três marcadores tubulares citados previu com precisão o agravamento da função renal nos pacientes com ICD.^38^

Estima-se que microalbuminúria esteja presente em 20% a 30% dos pacientes com IC. Dois estudos demonstraram associação com mortalidade em pacientes com micro ou macroalbuminúria comparados àqueles com excreção normal de albumina.^39^

Na SCRA, o paciente piora clinicamente e desenvolve oligúria, apesar dos níveis elevados de peptídeos natriuréticos, que têm sabidamente efeito diurético. É importante lembrar que o NT-proBNP é reduzido em pacientes submetidos a hemodiálise por membranas de alto fluxo.^40^

O ST2 (do inglês, *Suppression of Tumorigenicity 2* ) é um biomarcador de congestão menos influenciado pela função renal do que o NT-ProBNP e pode acrescentar informação diagnóstica e prognóstica.^41^

### Métodos de imagem no diagnóstico da síndrome cardiorrenal aguda

O exame de imagem com avaliação das ondas de fluxo venoso e arterial renal pode sinalizar um agravamento da função antes de ocorrer um aumento da creatinina sérica, fornecendo uma avaliação viável e não invasiva da hemodinâmica renal.^42 , 43^

### Implicações prognósticas da síndrome cardiorrenal aguda

A SCRA está associada com maior mortalidade por todas as causas e cardiovascular em curto e longo prazos, tempo de hospitalização prolongado,^10 , 11 , 44 - 46^ reinternações,^27 , 47^ progressão para os estágios da doença renal crônica^48^ e maiores custos com a saúde.^10^

A SCRA parece ser mais grave em pacientes com fração de ejeção do ventrículo esquerdo (FEVE) reduzida em comparação àqueles com FEVE preservada, atingindo uma incidência de 70% naqueles com choque cardiogênico.^49^ Além disso, o comprometimento da função renal é encontrado como um fator de risco independente para mortalidade em 1 ano em pacientes com IC aguda, incluindo aqueles com infarto do miocárdio com supradesnivelamento do segmento ST.^23^ Uma justificativa seria que um declínio agudo da função renal não atua simplesmente como um marcador de gravidade da doença, mas também acelera alterações cardiovasculares através da ativação de vias inflamatórias.^48^

Dois estudos mostraram que o risco de mau prognóstico permanece independentemente se a SCRA foi intermitente ou persistente^45 , 47^ e que mesmo discretas alterações da função renal podem alterar o risco de morte.^49^ Alguns estudos demonstraram que a SCRA persistente, quando comparada com a intermitente, apresenta pior prognóstico após a alta hospitalar e elevações transitórias da creatinina não se relacionaram com pior prognóstico.^50 - 52^

No estudo ADHERE,^9^ 59% dos homens e 68% das mulheres apresentaram disfunção renal moderada a grave no momento da hospitalização. Pacientes com piora da função renal durante a internação apresentaram maior mortalidade hospitalar. Pacientes cuja internação hospitalar é precipitada pela SCRA têm maior mortalidade hospitalar, maior tempo de hospitalização, mais reinternações e maiores taxas de mortalidade pós-alta comparados a pacientes com outros fatores precipitantes.^53 - 55^ A SCRA persistente em 1 ano após a alta mostrou-se um preditor significativo de mortalidade cardiovascular e mortalidade por todas as causas.^56^

Pelo menos um quarto dos pacientes hospitalizados com ICD pode desenvolver SCRA, dependendo do critério diagnóstico utilizado. Entre os pacientes com IC hospitalizados, um aumento na creatinina sérica é um dos mais importantes preditores de sobrevida^10 , 57^ e a mortalidade aumenta progressivamente com o incremento da creatinina sérica.^11 , 27 , 58 , 59^

Nem todas as alterações da função renal têm a mesma relevância prognóstica. O aumento da creatinina sérica concomitantemente com melhora dos sintomas e perda de peso não está associado a um desfecho desfavorável.^60^ A IRA indica que ocorreu lesão renal, que pode ser reversível ou não, enquanto que a piora dos marcadores da função renal pode representar um declínio funcional da TFG sem relação direta com um desfecho adverso.^61^

A SCRA intermitente reflete uma redução reversível na TFG e parece menos prejudicial do que a SCRA persistente. Paradoxalmente, nos casos de SCRA na admissão, a diminuição da creatinina durante a hospitalização pode estar associada a desfechos adversos.^28 , 53 , 62^ Considerando a congestão renal como o principal mecanismo fisiopatológico da SCRA, espera-se um efeito benéfico dos diuréticos no prognóstico. Uma análise *post hoc* do ensaio clínico DOSE^63^ mostrou que a melhora da função renal quando associada ao tratamento inadequado da congestão associou-se com pior prognóstico.

Outros estudos mostraram que, na terapêutica com diuréticos e hemoconcentração, a piora da função renal tem menor impacto prognóstico do que em pacientes com congestão persistente e ausência de hemoconcentração.^28 , 64^ Esse achado deve-se em parte a fatores de confundimento na avaliação da creatinina sérica. No contexto do tratamento da congestão, a elevação da creatinina sérica pode resultar de outros mecanismos independentes da redução da TFG, como a hemoconcentração que reduz a distribuição da creatinina. Essa alteração renal é inofensiva e transitória, denominada pseudo-IRA. O conceito de pseudo-IRA pode explicar porque os biomarcadores de lesão tubular foram pobres preditores de SCRA, posto que em estudos anteriores não houve a distinção entre IRA e pseudo-IRA.^62 , 65^ Durante a terapêutica diurética agressiva, o aumento da creatinina sérica ocorreu em 22% dos pacientes com ICD sem aumento de biomarcadores, sugerindo uma proporção potencialmente alta de pseudo-IRA.^65^

Não é fácil determinar se a terapêutica é eficaz e a pseudo-IRA pode induzir a descontinuação inapropriada do tratamento. É importante avaliar os parâmetros clínicos de perfusão, débito urinário, perda de peso e hemoconcentração. Além disso, os biomarcadores são promissores para orientar a terapia.^66^ A mensuração do débito cardíaco e de outros parâmetros hemodinâmicos pode ajudar a garantir uma terapia diurética adequada e direcionada^67^ e permitir um melhor entendimento do quadro de SCRA.

## Conclusões

As diferentes referências de creatinina sérica basal limitam a capacidade de fazer comparações precisas entre estudos e alteram as estimativas de diagnóstico da SCRA, superestimando ou subestimando.

Os autores deste artigo sugerem que os critérios de SCRA devem ser revisados para que se inclua o diagnóstico de SCRA na admissão hospitalar. A creatinina de referência deve refletir a função renal basal antes do início da IRA.
