# Supplementary material for: Síndrome Cardiorrenal Aguda: Qual Critério Diagnóstico Utilizar e sua Importância para o Prognóstico?
Source: Arq Bras Cardiol. 2020 Jul 28;115(1):127–33. [Article in Portuguese] doi: 10.36660/abc.20190207 (PMC8384316; doi:10.36660/abc.20190207)
Supplement: Translation

# Translation: Acute Cardiorenal Syndrome: Which Diagnostic Criterion to Use And What is its Importance for Prognosis?

**Authors:** Andréa de Melo Leite, Bruno Ferraz de Oliveira Gomes, André Casarsa Marques, João Luiz Fernandes Petriz, Denilson Campos de Albuquerque, Pedro Pimenta de Mello Spineti, Antonio José Lagoeiro Jorge, Humberto Villacorta, Wolney de Andrade Martins

**Affiliations:** 1 Brazil Universidade Federal Fluminense - Faculdade de Medicina - Pós-graduação em Ciências Cardiovasculares, Niterói, RJ – Brazil; 2 Brazil Rede D’Or São Luiz, Rio de Janeiro, RJ – Brazil; 3 Brazil Instituto D’Or de Pesquisa e Ensino, Rio de Janeiro, RJ – Brazil; 4 Brazil Universidade do Estado do Rio de Janeiro, Rio de Janeiro, RJ – Brazil

**Keywords:** Cardiorenal Syndrome, Renal Insufficiency, Creatinine, Prognosis, Heart Failure, Systematic Review

## Abstract

The absence of a consensus about the diagnostic criteria for acute cardiorenal syndrome (ACRS) affects its prognosis. This study aimed at assessing the diagnostic criteria for ACRS and their impact on prognosis. A systematic review was conducted using PRISMA methodology and PICO criteria in the MEDLINE, EMBASE and LILACS databases. The search included original publications, such as clinical trials, cohort studies, case-control studies, and meta-analyses, issued from January 1998 to June 2018. Neither literature nor heart failure guidelines provided a clear definition of the diagnostic criteria for ACRS. The serum creatinine increase by at least 0.3 mg/dL from baseline creatinine is the most used diagnostic criterion. However, the definition of baseline creatinine, as well as which serum creatinine should be used as reference for critical patients, is still controversial. This systematic review suggests that ACRS criteria should be revised to include the diagnosis of ACRS on hospital admission. Reference serum creatinine should reflect baseline renal function before the beginning of acute kidney injury.

## Introduction

Heart failure (HF) is a clinical challenge and a growing epidemiological problem worldwide, with high morbidity and mortality.^1^ In the ARIC study,^2^ the case fatality rates within 30 days, 1 year and 5 years from hospitalization due to HF were 10.4%, 22.0% and 42.3%, respectively. The I Brazilian Registry of Heart Failure (BREATHE),^3^ an observational study with 1263 patients from different Brazilian regions, has shown in-hospital mortality of 12.6%.

Cardiorenal syndrome, defined as kidney injury caused by HF, was first described in 1951^4^ and categorized into five types in 2008 ( Table 1 ).^5^ Type 1 cardiorenal syndrome or acute cardiorenal syndrome (ACRS) is characterized by acute kidney injury (AKI) caused by decompensated HF (DHF). Some authors refer to ACRS as acute worsening of renal function in patients with HF, which is a frequent condition, present in 11% to 40% of hospitalizations due to DHF.^6 , 7^

**Table 1:** – Types of cardiorenal syndrome

Type	Name	Mechanism
1	Acute CRS	AKI induced by acute cardiac dysfunction
2	Chronic CRS	Progressive CKF secondary to chronic HF
3	Acute renocardiac syndrome	Acute HF precipitated by AKI
4	Chronic renocardiac syndrome	HF secondary to CKF
5	Secondary CRS	Myocardial and renal dysfunction due to systemic diseases

*CRS: cardiorenal syndrome; AKI: acute kidney injury; CKF: chronic kidney failure; HF: heart failure.*

*Source: Di Lullo et al.^40^*

Worsening of renal function is defined as an absolute increase in serum creatinine by 26.5 μmol/L, equivalent to 0.3 mg/dL, and/or a 25% increase in creatinine or a 20% decrease in glomerular filtration rate (GFR).^8^ The criterion of absolute 0.3-mg/dL increase in creatinine has been adopted by most authors as the cutoff point to define ACRS.

The North American ADHERE registry^9^ is an observational study with more than 100,000 patients hospitalized with DHF, 35% of whom had moderate to severe renal dysfunction.

Worsening of renal function occurs in 30% to 50% of patients with DHF, depending on the definition used, and is associated with longer length of hospital stay, as well as higher health care costs and mortality.^10 - 14^ However, the absence of a consensus definition of ACRS contributes to the lack of clarity in its diagnosis and treatment.^15^ The choice of reference serum creatinine to anchor the diagnostic criteria for ACRS is a challenge. Ideally, reference serum creatinine should reflect the baseline renal function before AKI begins. Most of the time, however, that information is not available, leading to the use of surrogate reference values, which can result in misclassification of ACRS regarding its diagnosis and severity.^16^

## Methods

This review was conducted according to the Preferred Reporting Items for Systematic Reviews and Meta-Analyses (PRISMA) methodology.^17^ Data search in the MEDLINE, EMBASE and LILACS databases included the full texts of original publications, such as clinical trials, cohort studies, case-control studies and meta-analyses, issued from January 1998 to June 2018, written in English, Spanish and Portuguese. The database search was conducted with the following descriptors: ( *cardiorenal syndrome* ) OR *(worsening renal function)* AND ( *heart failure* ) AND ( *diagnosis* ) AND ( *prognosis* ).

This study used the PICO (Population, Intervention, Control and Outcome) framework for literature search and reviewed the diagnostic criteria for ACRS and their prognostic implication for the outcomes ‘in-hospital mortality’, ‘mortality after hospital discharge’, and ‘length of hospital stay’. Case reports and experimental animal models were excluded.

## Results

Regarding database search, 368 abstracts met the established criteria. Other 9 articles were retrieved in other sources, while 278 duplicate abstracts were removed. Of the 99 abstracts left, 61 were selected, 35 of which were excluded for not meeting the previously established criteria (PICO). The full text of the resulting 26 articles was then assessed regarding their scientific quality, and 4 articles were excluded for not meeting the criteria. The remaining 22 articles were analyzed in this study ( Figure 1 ).

**Figure 1:** – *Flowchart of the studies assessed (PRISMA methodology^17^).*

### Temporal classification of acute cardiorenal syndrome

Studies with access to pre-admission serum creatinine have revealed AKI in one third of the patients presenting to the emergency department,^18^ while 50% of patients have been reported to develop AKI within the first 48 hours from admission. Tayaka et al.,^19^ in a study comparing renal function changes up to the fourth day of hospitalization with those from the fifth day onward, have reported higher mortality within 1 year from hospital discharge in patients with late renal injury. A *post hoc* analysis of the Pre-RELAX study has shown that the drop in systolic blood pressure in the first 48 hours of vasodilator therapy was an independent predictor of AKI up to the fifth day of hospitalization.^20^ Those results suggest that therapy-related reduction in renal perfusion pressure is one of the major mechanisms leading to AKI in the first days of hospitalization.

Acute cardiorenal syndrome can be classified into intermittent or persistent. Intermittent ACRS occurs when serum creatinine levels vary during hospitalization with a reduction in its values up to discharge time. Persistent ACRS occurs when either creatinine elevation or GFR decrease persist up to discharge time.^21 , 22^

### Incidence of acute cardiorenal syndrome

Studies have shown a large variation in the incidence of ACRS, whose estimates range from 19% to 45%. That variation can be attributed to differences among the studies regarding their diagnostic criteria, their inclusion and exclusion criteria, their sample sizes, and the clinical findings of the populations studied. Most studies involve retrospective, secondary and/or *post hoc* analyses of large databases^10 - 12 , 23 - 25^ or clinical trials of drug therapy.^26^

### Diagnostic criteria for acute cardiorenal syndrome

The first study to assess the impact of worsening renal function on the elderly admitted with DHF, published in 2000, adopted the 0.3-mg/dL increase in creatinine as the criterion.^10^ Another study has shown that 0.1-mg/dL increases in creatinine during hospitalization were associated with higher in-hospital mortality and longer length of hospital stay. In addition, that study reported that creatinine increase ≥0.3 mg/dL had higher sensitivity and specificity to predict both death (81% and 62%, respectively) and length of hospital stay longer than 10 days (64% and 65%, respectively).^11^

Absolute creatinine increase by 0.3 mg/dL has been adopted by most authors as the criterion defining ACRS.^27^ Some authors, however, disagree, because that criterion does not consider the previous degree of renal dysfunction, and they suggest using one of three different classifications to define AKI,^28^ which, however, are not specific for DHF and have been developed to define and classify AKI in different clinical scenarios.

The RIFLE (Risk, Injury, Failure, Loss and End-stage renal disease) classification^29^ was proposed in 2004 to define and stratify the severity of AKI, which is determined by the most altered parameter (creatinine variation, GFR and urine output).

The classification proposed by the Acute Kidney Injury Network (AKIN)^30^ excludes the stages of ‘renal function loss’ and ‘end-stage renal disease’, as well as the ‘GFR-based criteria”. Staging should be performed after correcting the patient’s blood volume, excluding urinary tract obstruction, and considering the most altered criterion. In 2012, the *Kidney Disease – Improving Global Outcomes* (KDIGO)^31^ group proposed a classification modifying the previous one by adding to its third stage GFR reduction to values below 35mL/min/1.73m^2^in patients under the age of 18 years and excluding the need for the minimum 0.5-mg/dL increase for patients with creatinine greater than 4 mg/dL.

A cohort study assessing 637 hospitalizations due to DHF with 30-day and 1-year follow-up assessments has compared the diagnostic criterion of creatinine increase ≥0.3 mg/dL with those from KDIGO, RIFLE and AKIN regarding prediction of the following outcomes: ‘death’, ‘readmission due to HF’ or ‘initiation of dialysis’. Regarding the ability to determine adverse events, the four criteria performed similarly. The benefit of using the AKI classification systems (RIFLE, AKIN, KDIGO) is the possibility to identify patients with more severe AKI who will have adverse events in 30 days and 1 year.^32^Table 2 summarizes the different diagnostic criteria for AKI found in the literature.

**Table 2:** – RIFLE34, AKIN35, KDIGO36 and WRF11 criteria for definition of AKI

Criteria	WRF	RIFLE		AKIN	KDIGO
Years	2002	2004		2007	2012
Classification	sCr increase	sCr increase	GFR decrease	sCr increase	sCr increase
Stage 1 / Risk	≥ 0.3 mg/dL	≥ 1.5x bCr	≥ 25%	> 1.5-1.9x bCr or ≥ 0.3 mg/dL	≥ 1.5x bCr or ≥ 0.3 mg/dL
Stage 2 / Injury	-	≥ 2x bCr	≥ 50%	> 2-2.9x bCr	≥ 2x bCr
Stage 3 / Failure	-	≥ 3x bCr	≥ 75%	≥ 3x bCr	≥ 3x bCr
	-	Or sCr ≥ 4mg/dL and a 0.5-mg/dL increase		Or sCr ≥ 4mg/dL and a 0.5-mg/dL increase or initiation of dialysis	Or sCr ≥ 4mg/dL
Minimum time for AKI to occur	sCr can increase at any time during hospitalization	sCr changes over 1-7 days for more than 24 h		Acute sCr changes within a 48-h period during hospitalization	SCr changes ≥ 1.5x bCr within 7 days, or 0.3-mg/dL minimum increase in sCr within a 48-h period

*WRF, worsening renal function; AKI, acute kidney injury; sCr, serum creatinine; GFR, glomerular filtration rate; bCr, baseline creatinine.*

*Source: Adapted from Roy et al.^32^*

The most used diagnostic criterion is serum creatinine increase by at least 0.3 mg/dL or 25% in the first five days of hospitalization, which differs from the current KDIGO definition for AKI.^33^ In addition, the definition of worsening renal function does not include AKI on admission, which is associated with mortality and cardiovascular events.^34^

Common approaches to the ACRS diagnosis include the use of the following reference values of baseline creatinine, from which the creatinine increase defines ACRS: a) serum creatinine on admission; b) the lowest creatinine during hospitalization; c) serum creatinine levels of other hospitalizations; or d) outpatient measurements of serum creatinine. The original criteria of the RIFLE classification do not specify the reference creatinine but recommend its calculation from an estimated GFR of 75mL/min/1.73m^2^. Other approaches include the assessment of creatinine variation in the first 48 hours from admission, to reduce the need for the pre-hospital value (AKIN), and the lowest serum creatinine during hospitalization, when the outpatient measurement of serum creatinine is absent (KDIGO).^35^

Siew et al.,^36^ studying 4,863 in-hospital patients, have assessed three reference values of baseline creatinine: MDRD ( *Modification of Diet in Renal Disease* ), serum creatinine on admission, and the lowest creatinine during hospitalization. The use of MDRD and *nadir* creatinine inflated the incidence of AKI by about 50%; in contrast, the use of the admission value underestimated it by 46%. The use of the admission creatinine value as reference has the lowest sensitivity for the diagnosis of AKI acquired at the hospital and does not include the diagnosis of pre-admission AKI. Some authors consider as reference the pre-admission creatinine (outpatient measurement) when available, but only some of them have defined the time of maximum validity of the outpatient measurement up to admission. However, the outpatient value of creatinine is rarely available.

The RIFLE classification^29^ does not define specifically reference baseline creatinine. The most recent AKI criterion, KDIGO, recommends the lowest serum creatinine during hospitalization to be used as reference.^35^ Few studies have considered baseline renal function correlated with increasing creatinine during the AKI episode.

### The use of biomarkers to characterize acute cardiorenal syndrome

Although creatinine is the pillar of the diagnosis of ACRS, it has limitations as a marker of renal function, mainly in critical patients. Its serum level is influenced by factors, such as sex, age, body weight, and muscle mass. In addition, creatinine increases only 24 hours after kidney injury and its concentration does not increase significantly until half of renal function is impaired. Thus, creatinine is considered a slow marker of AKI.^37^ The definition of baseline creatinine in critical patients is controversial because those patients have nutritional alterations, muscle mass loss and fluid overload.

Useful biomarkers are those with clinical applicability and a recognized role in the pathophysiology of ACRS. The search for more reliable biomarkers for the early diagnosis of ACRS is encouraged, and kidney injury molecule-1 (KIM-1), neutrophil gelatinase-associated lipocalin (NGAL), interleukin 18 (IL-18), and cystatin C (Cys-C) are some of the new markers of kidney injury targeted in studies. However, none of the three renal tubular markers cited could predict accurately worsening renal function in patients with DHF.^38^

Microalbuminuria is estimated to be present in 20% to 30% of patients with HF. Two studies have shown association with mortality in patients with micro- or macroalbuminuria as compared to those with normal albumin excretion.^39^

The clinical condition of patients with ACRS deteriorates and they develop oliguria, despite the high levels of natriuretic peptides, which are known to have a diuretic effect. It is worth noting that NT-proBNP levels are reduced in patients undergoing hemodialysis with high-flux membrane.^40^

The suppression of tumorigenicity-2 (ST2), a biomarker of congestion less influenced by renal function than NT-ProBNP, might be helpful for diagnostic and prognostic information.^41^

### Imaging methods for the diagnosis of acute cardiorenal syndrome

Renal imaging with assessment of the waves of venous and arterial renal flows can signal worsening renal function before serum creatinine levels increase, providing a feasible and non-invasive assessment of renal hemodynamics.^42 , 43^

### Prognostic implications of acute cardiorenal syndrome

Acute cardiorenal syndrome is associated with the following: higher all-cause and cardiovascular mortality in the short and long run; prolonged length of hospital stay;^10 , 11 , 44 - 46^ re-admissions;^27 , 47^ progression to chronic kidney disease;^48^ and higher health care costs.^10^

Acute cardiorenal syndrome is apparently more severe in patients with reduced left ventricular ejection fraction (LVEF) as compared to those with preserved LVEF, reaching the incidence of 70% in patients with cardiogenic shock.^49^ In addition, renal function impairment is an independent risk factor for 1-year mortality in patients with acute HF, including those with ST-elevation myocardial infarction.^23^ Moreover, an acute decline in renal function not only acts as a marker of disease severity, but also speeds cardiovascular alterations up by activating inflammatory pathways.^48^

Two studies have shown that the risk of poor prognosis remains independently of the ACRS type (intermittent or persistent)^45 , 47^ and that even mild renal function changes can alter the risk of death.^49^ Some studies have shown that persistent ACRS, as compared to intermittent ACRS, has worse prognosis after hospital discharge and that transient creatinine elevations did not relate to worse prognosis.^50 - 52^

In the ADHERE study,^9^ 59% of the men and 68% of the women had moderate to severe renal dysfunction on admission, and those with worsening renal function during hospitalization had higher in-hospital mortality. Patients whose hospitalization is precipitated by ACRS have higher in-hospital mortality, longer length of hospital stay, more re-admissions and higher mortality rates after discharge as compared to patients with other precipitating factors.^53 - 55^ Persistent ACRS within 1 year from hospital discharge was a strong predictor of cardiovascular and all-cause mortality.^56^

At least one fourth of the patients hospitalized with DHF can develop ACRS, depending on the diagnostic criterion used. Among patients hospitalized with HF, serum creatinine increase is one of the major predictors of survival,^10 , 57^ and mortality increases progressively as serum creatinine increases.^11 , 27 , 58 , 59^

Not all changes in renal function have the same prognostic relevance. Serum creatinine elevation concomitantly with symptom improvement and body weight loss is not associated with an unfavorable outcome.^60^ The presence of AKI indicates that a reversible or irreversible kidney lesion has occurred, while worsening renal function markers can represent a functional decline in GFR not directly related to an adverse outcome.^61^

Intermittent ACRS reflects a reversible reduction in GFR and seems less harmful than persistent ACRS. Paradoxically, in cases of ACRS on admission, the decrease in creatinine during hospitalization can be associated with adverse outcomes.^28 , 53 , 62^ Considering renal congestion as the major pathophysiological mechanism of ACRS, diuretics are expected to have a beneficial effect on prognosis. A *post hoc* analysis of the DOSE trial^63^ has shown that renal function improvement when associated with inadequate decongestive strategies had a worse prognosis.

Other studies have shown that, with diuretic therapy and hemoconcentration, worsening renal function has a lower impact on prognosis than in patients with persistent congestion and no hemoconcentration.^28 , 64^ Those findings are partially due to confounding factors in serum creatinine assessment. In the context of measures of decongestion, the increase in serum creatinine can result from other mechanisms regardless of GFR reduction, such as hemoconcentration that reduces the distribution of creatinine. That renal change is harmless and transient, and named pseudo-AKI. The concept of pseudo-AKI can explain why biomarkers of tubular lesion were poor predictors of ACRS, considering that previous studies have made no distinction between AKI and pseudo-AKI.^62 , 65^ During aggressive diuretic therapy, serum creatinine increased in 22% of the patients with DHF without increase in biomarkers, suggesting a potentially high proportion of pseudo-AKI.^65^

It is not easy to determine whether the therapy is effective and pseudo-AKI can induce inadequate discontinuation of treatment. It is worth assessing the clinical parameters of perfusion, urine output, body weight loss and hemoconcentration. In addition, biomarkers seem to be good to guide therapy.^66^ Measuring cardiac output and other hemodynamic parameters can help ensure an adequate and directed diuretic therapy,^67^ in addition to enabling better understanding of ACRS.

## Conclusions

The different references of baseline serum creatinine limit the capacity of accurate comparisons between studies and interfere with the estimates of ACRS diagnosis, overestimating or underestimating it.

This study suggests that the ACRS criteria should be revised to include the diagnosis of ACRS on hospital admission. Reference creatinine should reflect baseline renal function before AKI begins.
